# Tolerance to a Diet of Toxic *Microcystis aeruginosa* in *Caenorhabditis elegans*

**DOI:** 10.3390/toxins17030109

**Published:** 2025-02-27

**Authors:** Jordan Balson, Jeffrey R. Boudreau, Ian D. Chin-Sang, Yuxiang Wang, Daniel D. Lefebvre

**Affiliations:** Department of Biology, Queen’s University, 116 Barrie St., Kingston, ON K7L 3J9, Canada; 14jlb11@queensu.ca (J.B.); boudreauj@queensu.ca (J.R.B.); chinsang@queensu.ca (I.D.C.-S.); yuxiang.wang@queensu.ca (Y.W.)

**Keywords:** cyanobacteria, bioremediation, nematodes, *Caenorhabditis elegans*, *Microcystis aeruginosa*, microcystins, toxin tolerance

## Abstract

Reported incidences of cyanobacterial harmful algal blooms (CHABs) are increasing across the world due to climate change and nutrient loading, dominating freshwater ecosystems and producing dangerous cyanotoxins that cause ecological damage. *Microcystis aeruginosa* is one of the most common species of cyanobacteria; it produces hepatotoxic and neurotoxic microcystin-LR. The ecological and human impact of algal blooms is immense, and traditional CHAB remediation methods are not always adequate in eutrophic regions such as Lake Erie in North America. As a result, a proactive, targeted approach is needed to bioremediate cyanobacteria in their pre-colonial stages. Nematodes, such as the model organism *Caenorhabditis elegans*, are potential candidates for bioremediating cyanobacteria such as *M. aeruginosa*. *C. elegans* have metabolic pathways that could detoxify microcystin-LR and enable tolerance to cyanobacteria in nature. We analyzed *C. elegans* health and fat accumulation on a diet of toxic *M. aeruginosa* and found that *C. elegans* can ingest, digest, metabolize, and survive off of this diet. The mean lifespans of the worm populations were only slightly different at 20.68 ± 0.35 (mean ± S.E.M) and 17.89 ± 0.40 when fed *E. coli* and toxic *M. aeruginosa*, respectively. In addition, a diet of toxic *M. aeruginosa* compared to *E. coli* did not have any significant impact on *C. elegans* pharyngeal pumping (304.2 ± 9.3 versus 330.0 ± 10.4 pumps/min), dauer response (86.3 ± 1.0 versus 83.65 ± 1.0% in dauer), mobility (209.25 ± 7.0 versus 210.15 ± 4.4 thrashes/min), or SKN-1 expression based on SKN1::GFP fluorescence measurements. Overall, a diet of toxic *M. aeruginosa* was able to sustain *C. elegans* development, and *C. elegans* was tolerant of it. These results suggest that *C. elegans* and similar nematodes could be viable candidates for cyanobacterial bioremediation.

## 1. Introduction

Cyanobacterial harmful algal blooms (CHABs) are increasing in occurrence and severity worldwide [1]. This increase is mainly due to climate change and eutrophication, the latter caused by nitrogen and phosphorus leaching from increased agricultural activity and urban and industrial development [2]. Cyanobacteria are able to enhance their growth rates, aggregate, and amplify their toxic effects in their bloom form [3,4,5]. The mechanisms that control cyanobacterial toxicity are not completely understood; however, it has been observed that when the cells are injured or die, their previously cell-bound or intracellular cyanotoxins are released [4,6]. This would also occur during their breakdown during digestion by animals. CHABs disrupt their ecosystems in many ways in addition to cyanotoxin release; they cause increased turbidity, with thick blooms reducing sunlight penetration to other autotrophic life, and hypoxia when their excess cellular biomass dies and becomes degraded [2,7]. In humans, CHAB exposure has been linked to gastrointestinal disorders, hypertension, skin issues, and flu-like symptoms such as fever and chills [4].

Controlling CHABs and their cyanotoxins has historically proven to be a difficult task. Traditionally, CHABs have been controlled through post-bloom control, such as algaecide applications [2] that have dangerous side effects to non-target organisms and anoxic effects from rapid CHAB collapse [2,8,9]. CHAB control methods also include preventative measures such as nutrient load budgeting and watershed management to prevent eutrophication. However, these strategies usually require adequate identification and control of multiple nutrient sources and long timeframes for implementation [2,8]. In all, these approaches, even when combined, have proven to not always be adequate and can be ineffective in extremely eutrophic bodies of water [10]. Additionally, cyanobacteria can descend in the water column during overwintering, where they form benthic mats, enabling them to subsequently ascend when warmer conditions return so they can enter their disruptive colonial phase [11]. Overall, more research is required to develop CHAB treatment methods that prevent cyanobacterial infiltration and damage to the water column and benthos ecosystems [2,8].

*Microcystis* is a genus of cyanobacteria that produces cyanotoxins called microcystins [4,12]. *Microcystis aeruginosa* is the overall most common toxigenic, fresh-water bloom-forming species of cyanobacteria [4,13]. It is dominant in CHABs worldwide and known to be involved in most incidences of animal and human poisoning.

Cyanotoxin exposure can have a range of effects in animals. Acutely, it can cause contact irritation, gastrointestinal issues, paralysis, aberrant feeding behaviors, and shortened longevity. Chronically, exposure can cause metabolic alterations, behavioral modifications, and decreases in growth and in biological fitness [4,13]. Of these toxins, microcystins are the most investigated; they are cyclic peptides and are classified as hepatotoxins, neurotoxins, and strong tumor promoters [13,14]. One widespread congener is microcystin-LR, (MC-LR) which is a leucine/arginine variant that demonstrates high toxicity, resulting in high moribundity from oral ingestion [15]. The stability of MC-LR enables it to accumulate in bodies of water, prolonging its damage to ecosystems [12].

*Caenorhabditis elegans* is a model nematode that is used as an indicator species of this group of organisms [16]. It has a short lifespan of 2–3 weeks, and the determination of its longevity is through the interplay of many different biochemical signaling cascades [17]. Mobility is a reliable predictor of vigor that correlates well with increased survival; as a result, thrashing assays are an established indicator of *C. elegans* general health [16,18].

*C. elegans* is bacterivorous and has been observed to consume decomposing matter. It has dietary preferences, actively choosing foods that are more nutritious. Cyanobacteria have also been found to be present in the microbiome of *C. elegans*, suggesting that they ingest it in nature [19]. Their feeding behavior can be easily monitored through their pharyngeal pumping mechanism, where the bacteria are captured, pulled back, and propelled into the lumen of the worm’s intestinal tract [20]. The pharyngeal pumping results from the combination of two motions, the pumping of the pharynx and an isthmus peristalsis, which moves the food along the isthmus through peristaltic waves. From there, the food is broken down with the cuticle grinder in the terminal bulb [21,22]. In addition to diet, *C. elegans* fat accumulation depends on the nematode’s developmental stage and exposure to stressors [20]. *C. elegans* respires through diffusion, and while it is predominantly terrestrial and in nature typically lives in soil, it can survive and be cultured in liquid environments [19,23,24]. When *C. elegans* is in its first larval stage, and there are unfavorable conditions such as chronic heat stress, the nematode can enter an alternative developmental path known as dauer diapause. This is a period of developmental arrest where metabolic changes cause the worm to live off of its fat reserves and stop feeding, but it can still demonstrate movement [20,25,26]. In dauer, the worms can tolerate several stressful conditions, such as desiccation, hypoxia, starvation, and heat. Once environmental conditions become favorable, the organisms proceed directly to reproductive adults [25,26].

Microcystin tolerance and detoxification is thought to be controlled by stress response pathways like the phase II detoxification system. For example, in a study by Wang and colleagues [27], phytoplanktivorous tilapia that strongly tolerate microcystin exposure responded by increasing mRNA levels for enzymes involved in oxidative stress and the phase II detoxification system. Although the potential detoxification ability of *C. elegans* is not completely elucidated, it might also involve the phase II detoxification system [28]. During xenobiotic stress, the skinhead-1 regulatory protein (SKN-1) activates transcription of its target genes. Doing so enables the system’s enzymes to scavenge free radicals, catalyze reactions that increase xenobiotic solubility and excretion, and synthesize glutathione, leading to toxin break down [28,29,30]. However, this would also demonstrate that *C. elegans* experiences severe stress in the presence of MC-LR. More importantly, a lack of SKN-1 expression would indicate that the nematode model *C. elegans* is able to tolerate MC-LR and therefore would be a suitable candidate for *Microcystis* bioremediation.

Overall, a bioremediation system that removes cyanobacterial cells from the water column before they can reach their destructive colonial phase would be ideal. Control prior to severely toxic CHAB formation would be effective at lower than the MC-LR concentrations set for drinking water, which are 1.5 µg/L [11] and 1.6 µg/L [31] for Canada and the United States, respectively. Higher toxin concentrations indicate a severe bloom is taking place, which would probably be much less amenable to bioremediation.

Biological control systems, once implemented, are extremely advantageous, primarily due to their simplicity of application, high specificity, and effectiveness [32]. Other benefits include that they are environmentally safe, natural, widely accepted, integrated with other organisms in the ecosystem, and less expensive than traditional contamination cleaning methods [33]. Therefore, this study examines the possible use of nematodes in the biological control of pre-bloom *M. aeruginosa* by determining this toxic cyanobacterium’s effects on the health and longevity of the model organism *C. elegans*.

## 2. Results

Several indicators of health and longevity in *C. elegans* were investigated in response to consuming *M. aeruginosa* that produces microcystin-LR.

### 2.1. Presence of Microcystin-LR

**Figure 1:**
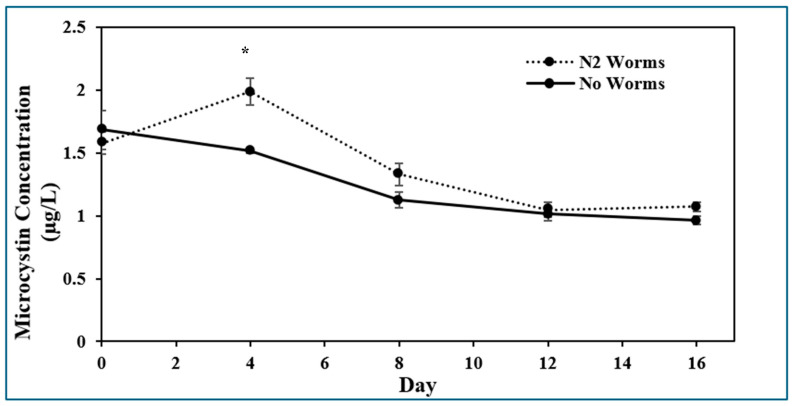
Microcystin-LR concentration in the media on plates seeded with partially lysed toxic *M. aeruginosa* in the presence and absence of *C. elegans*; * indicates significance (*p* < 0.05, X̄ *±* SE, n = 3).

Each day of sampling required new biological replicates because sampling at each time point for MC-LR destroyed the replicate; i.e., different biological replicates were resampled over the course of the experiment. In addition, the extraction procedure detailed in the Section 5 would have only extracted a proportion of the total MC-LR, and it is assumed that this proportion was consistent between samples. Therefore, the measurements as performed were expected to provide relative concentrations between treatments. ELISA assays showed that the concentration of MC-LR increased significantly after a 4-day exposure to *C. elegans* (Figure 1). Then, it decreased and was not significantly different from the worm-free treatment for the remaining sampling times up to 16 days.

### 2.2. Fat Accumulation

**Figure 2:**
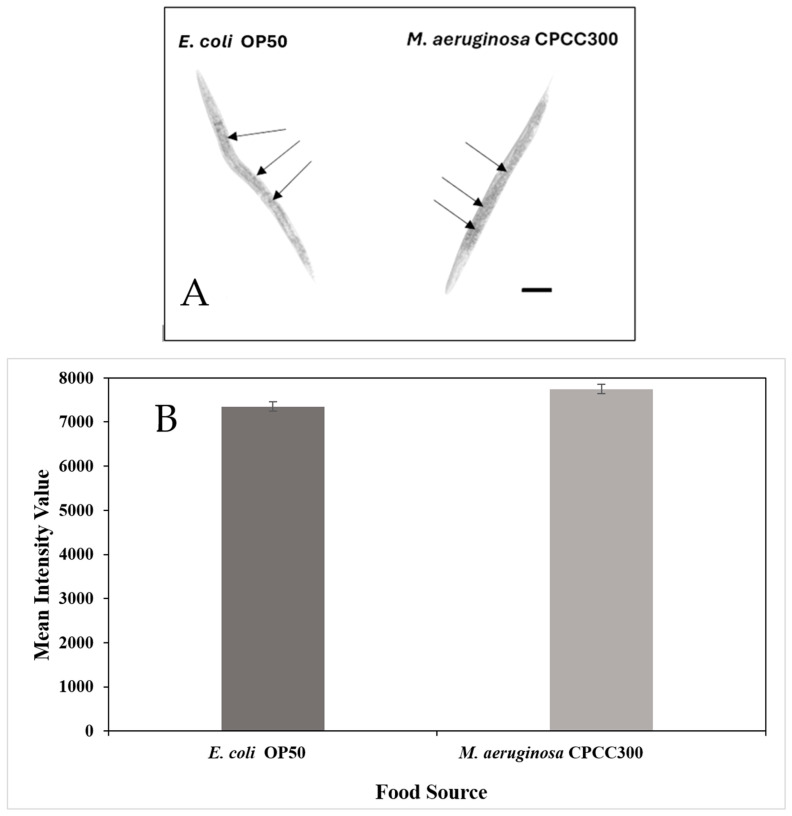
Quantification of storage fats in 4-day-old *C. elegans*. (**A**) Micrographs of nematodes after neutral lipid staining with Oil-Red O on different diets (Bar = 100 µm). (**B**) Relative amounts of staining for neutral lipids (X̄ *±* SE, n = 27).

The results of one of three independent experiments for fat accumulation is presented. There was a significant difference in the quantity of stored fat between the differently fed *C. elegans* (*p* = 0.012). The intensity value of stained fat of the nematodes on a diet of *M. aeruginosa* CPCC300 (7747.2 *±* 106.3) was higher than on a diet of *E. coli* OP50 (7350.1 *±* 105.4), indicating that they accumulated significantly more fat on the former diet (Figure 2; *p* = 0.01). Overall, this assay indicated that *C. elegans* can efficiently metabolize and accumulate fat from a diet of toxic *M. aeruginosa.*

### 2.3. Longevity Assay

**Figure 3:**
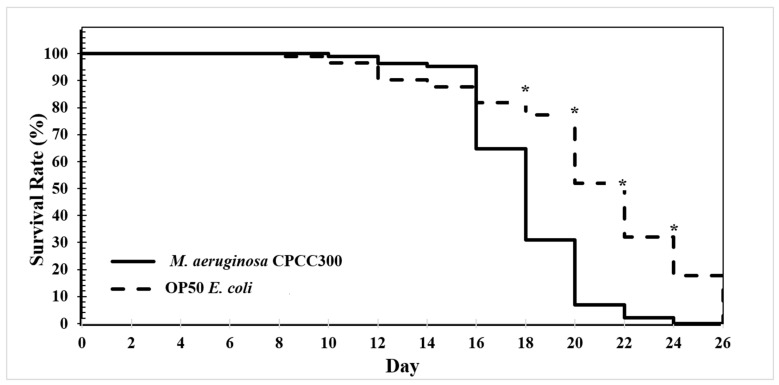
Longevity of N2 *C. elegans* on different diets. * indicates significant difference between the treatments at that time point (*p* < 0.95; X̄ *±* SE, n = 9).

For the longevity assay assessment of N2 *C. elegans*, survival rates on a diet of toxic *M. aeruginosa* and *E. coli* were performed (Figure 3). The mean and median lifespan of the worm populations per plate were 20.68 ± 0.35 and 20.6 days for OP50, respectively, and 17.89 ± 0.40 and 17.76 days for toxic *M. aeruginosa*, respectively. A two-tailed *t*-test of the mean population age at death gave a *p*-value of 7.95 × 10^−5^, indicating a significant difference. Overall, this shows that between the two treatments, the nematodes on a diet of *E. coli* maintained only slightly larger populations than those fed toxic cyanobacteria.

### 2.4. Stress Response Assay

Physiological responses including the percentage of worms in dauer response/temporary dormancy under chronic heat stress, mobility responses evaluated through thrashing, and feeding response evaluated through pharyngeal pumping were measured under the two different dietary treatments (Table 1). Two-tailed *t*-tests assuming homoscedastic variance indicated that there were no significant differences between the treatments for all of these tested parameters. Therefore, the presence of toxic cyanobacteria had no significant physiological effect.

### 2.5. SKN-1::GFP Expression

**Figure 4:**
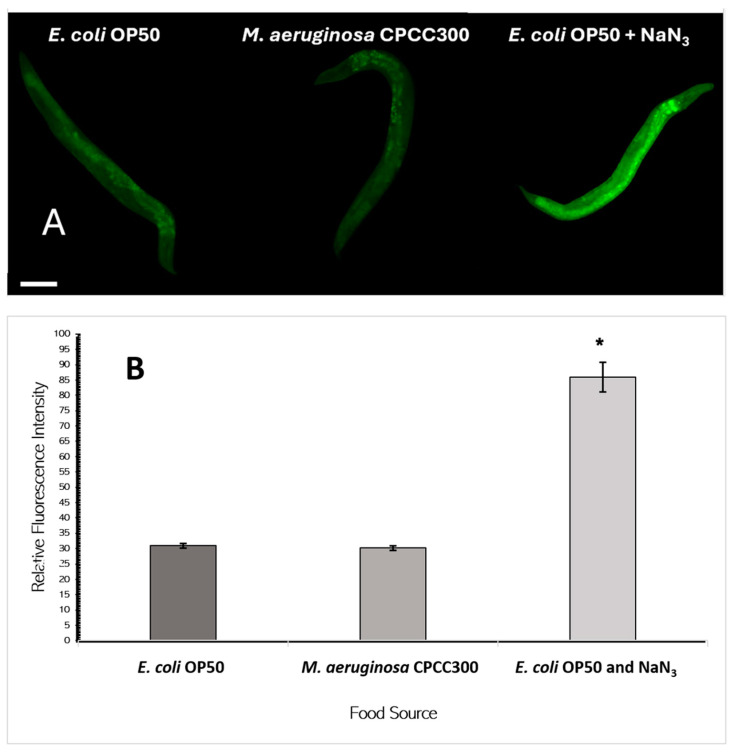
Determination of *SKN-1* expression as visualized through fluorescence intensity in *C. elegans* LD1 containing a SKN-1::GFP marker after 3 days in the different treatments. (**A**) Micrographs of nematodes on different diets (Bar = 100 µm); (**B**) relative amounts of fluorescence; * indicates significant difference from both other treatments (*p* < 0.95, X̄ *±* SE, n = 18).

SKN-1::GFP expression, an indicator of stress, was visualized as shown in Figure 4A. Fluorescence intensities of entire worms were measured and analyzed using a single-factor ANOVA. The relative fluorescence intensities when fed a diet of toxic *M. aeruginosa* (30.1 *±* 0.8) or *E. coli* OP50 (30.9 *±* 0.7) were both significantly less than the positive control of *E. coli* with sodium azide (86.0 *±* 4.80) but not significantly different from each other. Therefore, toxic cyanobacteria did not appear to cause stress in *C. elegans.*

## 3. Discussion

*C. elegans* was able to survive the presence of MC-LR that were released from *M. aeruginosa* through freezing and thawing (Figure 1). Furthermore, additional toxin was detected in the medium of the nematode treatment after 4 days, indicating that ingestion and digestion of *M. aeruginosa* caused significant additional MC-LR release from remaining intact cells and cell debris when compared to the absence of worms. It has been previously shown that lysed *M. aeruginosa* cells do discharge their intracellular toxins into the environment [6]; however, this phenomenon has not been documented from nematode activity. The MC-LR concentration as determined by analysis with ELISAs is relevant to environmental concerns where the maximum acceptable concentration has been set for drinking water at 1.5 µg/L [11] and 1.6 µg/L [31] for Canada and the United States, respectively.

The ability of *C. elegans* to survive to adulthood on a diet of toxic *M. aeruginosa* was similar to worms fed on *E. coli* OP50, and visualization of body fat confirmed that *C. elegans* were able to metabolize and accumulate fat from a diet of this cyanobacterium (Figure 2). In fact, the diet of *M. aeruginosa* resulted in slightly more fat accumulation than on a diet of OP50 *E. coli* (*p* = 0.012). This could be due to a variety of factors. *C. elegans* store their energy as triglycerides and primarily store fat in their intestinal and skin-like epidermal cells. These fat deposits increase in both number and size as the organism develops; however, fat deposition rate and their distribution can also vary substantially in response to environmental stressors [20]. The environmental pressures of a diet of toxic *M. aeruginosa* might have affected metabolism in such a way that enhances fat deposition. Additionally, the cyanobacteria may have a very nutritious fatty acid profile [5,34] that is efficiently metabolized and stored by *C. elegans*. Although *C. elegans* on a diet of toxic *M. aeruginosa* accumulated significantly more fat than the *C. elegans* on a diet of OP50 *E. coli*, both were similarly able to survive to adulthood. Therefore, the nematode was able to effectively metabolize toxic *M. aeruginosa.*

In the longevity assay, populations of *C. elegans* on a diet of toxic *M. aeruginosa* had significantly fewer individuals in their populations on days 18, 20, 22, and 24 (Figure 3), suggesting a negative impact on later-term longevity and overall health. This could be due to the cyanobacterial mucilage, which is a polysaccharide excretion that protects against grazing by zooplankton, among other roles [35,36,37]. It is, therefore, possible that it could also have a negative effect on *C. elegans* through consumption. Although high concentrations of MC-LR exposure have been observed to impact *C. elegans* survival and cause neurotoxic effects [38,39] the present study’s concentrations were two orders of magnitude lower than in these studies. Furthermore, it is known that *C. elegans* reproduction stops by 10–14 days of age [40], so shorter lifespans starting at day 18 should not ultimately affect the *C. elegans* biological fitness. Egg production could not be determined because of the opacity of the cyanobacterial lawn; however, normal progeny proceeded for at least 5 generations under both treatments.

For the dauer, thrashing, and pharyngeal pumping assays, there were no significant differences in phenotype expression between *C. elegans* on the two diets. Some of the impacts of exposure to high levels of MC-LR have been previously examined in *C. elegans* [41]. Exposure was found to affect both inhibitory and excitatory gamma-aminobutyric acid (GABA) receptors and to affect GABA transportation and localization. Because this neurotransmitter’s innervations control mobility, abnormal muscle contraction and relaxation resulted in decreased thrashing frequencies. Microcystin-LR exposure also decreased gene expression for the proteins necessary for GABAergic neurotransmission, causing neurobehavioral defects through probable neurotoxic effects on *C. elegans*. However, the present findings indicate that a diet of toxic *M. aeruginosa*, at much lower MC-LR concentrations (Figure 1), does not significantly affect *C. elegans* feeding behavior, mobility, or developmental stasis stress response; i.e., it does not affect these overall health-related characteristics.

SKN-1 is a central regulatory protein involved in detoxification and is involved in several stress response pathways, including oxidative, xenobiotic, or nutritional stress [29]. In the SKN-1::GFP expression experiment, a diet of *M. aeruginosa* compared to that of *E. coli* did not induce significantly greater expression of SKN-1, and it certainly did not induce SKN-1 expression to the level of the stress-induced positive control containing sodium azide (Figure 4). This suggests that a diet of *M. aeruginosa* does not invoke more of a stress response than the standard *C. elegans* diet. In the literature, MC-LR exposure has been observed to impact *C. elegans* survival and cause neurotoxic effects [38,39]. However, this was only observed at the higher concentrations of 250 to 500 μg/L. This is a drastically higher concentration than the present study’s ELISA assays indicated, where lysed toxic *M. aeruginosa* cells 4 days after *C. elegans* introduction had a MC-LR concentration of less than 2.0 μg/L. Although ultimately, SKN-1 still could be a central regulatory protein involved in microcystin tolerance, this demonstrates that the low MC-LR concentration released from a diet of toxic *M. aeruginosa* was not enough to induce a stress response.

It is worth noting that relatively high concentrations of MC-LR exposure in *C. elegans* have been shown to lead to a decreased lifespan, increased oxidative stress, and impaired reproduction and growth. However, these findings were at high concentrations of exogenous MC-LR (100 to 500 μg/L) itself [38,42] and not from exposure to natural toxin concentrations or from direct ingestion of *M. aeruginosa*.

Overall, CHABs are increasing in severity and prevalence across the world and are causing significant damage to human and animal health, and to the environment, resulting in substantial financial impacts [1,2,43]. As a result, a candidate that will digest *M. aeruginosa* off the benthos to prevent it from returning to the water column would be valuable. *C. elegans* is a ubiquitous model organism nematode that has been used to investigate many different fundamental aspects of biology [16]. This organism has been extensively investigated developmentally, physiologically, and genetically. It has stress response and detoxification pathways, such as the phase II detoxification system [29]. These facts, alongside the results of this study, show that the model nematode *C. elegans* is able to live and grow in the presence of MC-LR at environmentally realistic levels and on a diet of toxic *M. aeruginosa*.

## 4. Conclusions

This study revealed that *C. elegans* are able to accumulate fat and, as a result, are able to ingest, digest, and metabolize toxic *M. aeruginosa* at MC-LR concentrations emulating pre-bloom conditions up to that of drinking water advisory levels. This toxic diet had some minimal impact on longevity but does not appear to impact other health-indicator phenotypes, such as growth, mobility, feeding behavior, dauer developmental stasis stress response, expression of a major stress regulatory protein, and biological fitness. In all, nematodes that are capable of removing cyanobacteria from the water column through digestion would be beneficial to the health of the ecosystem, provided they are compatible with native organisms. Although the results of this study advocate that nematodes would be effective in the bioremediation of cyanobacteria, further investigations are necessary to demonstrate that *C. elegans* or possibly other nematode species would be successful in this role in natural habitats.

## 5. Materials and Methods

### 5.1. Bacterial Cultures

Toxic *Microcystis aeruginosa* CPCC300 was obtained from the Canadian Phycological Culture Centre (University of Waterloo, Waterloo, ON, Canada) and maintained in MA medium, adapted from Ichimura and colleagues [44] (0.05 g/L NaNO_3_, 0.1 g/L KNO_3_, 0.05 g/L Ca(NO_3_)_2_-4H_2_O, 0.04 g/L Na_2_SO_4_, 0.05 g/L MgCl_2_-6H_2_O, 0.05 g/L N-β glycerophosphate-5H_2_O, 0.005 g/L Na_2_EDTA, 0.0005 g/L FeCl_3_-6H_2_O, 0.005 g/L MnCl_2_-4H_2_O, 0.0005 g/L ZnCl_2_, 0.005 g/L CoCl_2_-6H_2_O, 0.0008 g/L Na_2_MoO_4_-2H_2_O, 0.02 g/L H_3_BO_3_, and 0.5 g/L Bicine; adjusted to pH 8.6 with 1M NaOH). Unless otherwise stated, all chemicals used in this study were of analytical grade or the highest purity available from Thermo Fisher Scientific (Ottawa, ON, Canada) or Milllipore Sigma (Oakville, ON, Canada). When required, approximately 50 mL of this stock culture was added to 1 L of MA medium and placed on a rotary shaker at 100 rpm and 25 °C under fluorescent lighting of 100 μmol/m^2^/s until the culture became dark green. The cyanobacteria were then centrifuged for 5 min at 1250× *g* to harvest the cells followed by adjustment to O.D._600_ of 3.0 before 100 µL were added to 55 mm nematode culture plates. *M. aeruginosa* was periodically checked for contamination through microscopic visualization.

*Escherichia coli* OP50 [45] was cultured on a rotary shaker at 200 rpm and 37 °C overnight in autoclaved 2xTY medium consisting of 16 g/L bacto tryptone, 10 g/L yeast extract, and 5 g/L NaCl [46]. The bacteria were then refrigerated until used, when 100 µL at O.D._600_ of 1.6 was added to the nematode culture plates. This is the standard amount of *E. coli* on which *C. elegans* is normally grown, and the amount of *M. aeruginosa* applied as food was based on the lower calorie contents of cyanobacteria compared to *E. coli* [47]. Egg production could not be determined because of the opacity of the cyanobacterial lawn; however, larval worms were observed.

### 5.2. Nematode Cultures

*C. elegans* strains were received from the Caenorhabditis Genetics Center (University of Minnesota, Twin Cities) and maintained on Nematode Growth Medium (NGM) agar plates [24] seeded with *E. coli* OP50 [48]. The Bristol wild-type strain N2 and the strain LD1 (*ldls7*; SKN-1::GFP; *pRF4*) were used in this study. The latter contains the genes for SKN1-1::GFP and a right roller phenotype marker in the N2 genetic background.

*C. elegans* were sterilized and their eggs harvested and prepared for all experiments through an L1 synchronization egg preparation as described by Porta-de-la-Riva and colleagues [49]. All experiments were performed at least three times.

### 5.3. Microcystin-LR Assay

Plates were seeded with lysed toxic *M. aeruginosa* CPCC300 to release MC-LR. Lysis was performed by aliquoting 3 mL of the cyanobacteria at O.D._600_ of 3.0 into 15 mL Falcon Conical Centrifuge Tubes (Thermo Fisher Scientific), freezing in liquid nitrogen, and thawing in a 30 °C water bath and repeating this twice [50]. The lysed cells were then vortexed for 2 min and stored at 4 °C. Then, 100 µL of this preparation was added to each plate containing 10 mL of NGM agar, followed by incubation at 20 °C in the presence or absence of *C. elegans*.

Samples to determine MC-LR concentrations were obtained by placing 1 mL deionized H_2_O onto the surface of each agar plate, followed by lightly swirling to ensure that the water touched the entire agar surface. The water was then collected through aspiration with a pipette, and this process was repeated two more times with the same water for each sample. Then 100 µL of worm-free supernatant obtained after the nematodes had settled to the bottom of the tube, was used for analysis. Because the sample procedure was destructive, each sample represented a distinct plate.

ELISA assays were then performed to determine the MC-LR concentrations by following the ABNOVA ELISA Microcystin-LR kit protocol (Abnova, Taipei, Taiwan). Based on the known concentrations of the standards and the absorbance values from the ELISAs, the sample concentrations were calculated from a generated standard curve. As a consequence of the addition of 1 mL of water, the total volume of each plate became 11.1 mL. Assuming uniform dilution of MC-LR, the estimated value obtained in the ELISA assays would be approximately 91% of that in the undiluted plates. The presented data takes this dilution factor into consideration.

### 5.4. Fat Accumulation

Synchronized L1 worms were plated on NGM agar plates seeded with either 100 µL toxic *M. aeruginosa* or 100 µL *E. coli* OP50, as described in Bacterial Cultures. The plates were cultured for 3 days at 20 °C, and then, the adult worms were stained following a protocol modified from O’Rourke and colleagues [51] as follows. Worms were collected in 1 mL of M9 [52] containing 0.1% Triton X-100, pipetted into a 1.5 mL microfuge tube, and then centrifuged for 1 min at 200× *g*. The supernatant was then discarded, and 1 mL of M9 was added to each centrifuge tube, which was then centrifuged for 1 min at 200× *g*. This wash was repeated three times, and the worms were then left in 100 μL M9 buffer. Then, 1 mL of 2x MRWB buffer was prepared fresh by adding 250 μL 16% paraformaldehyde to 750 μL MRWB stock solution (160 mM KCl, 40 mM NaCl, 14 mM Na_2_EGTA, 1 mM spermidine-HCl, 0.4 mM spermine, 30 mM Na-PIPES pH 7.4, and 0.2% β-mercaptoethanol). Worms were then fixed by adding 100 μL 2x MRWB buffer to the microfuge tube containing worms in 100 μL of M9 buffer, followed by incubation at room temperature for 1 h with gentle inversion every 15 min. Samples were centrifuged for 1 min at 200× *g* and the supernatant discarded. Then, 1 mL of M9 + 0.1% Triton X-100 was added, centrifuged again, and the supernatant removed; this washing step was repeated twice. The worms were then dehydrated by adding 1 mL of 60% 2-propanol and incubating for 15 min at room temperature in the dark. Oil Red O stock was freshly prepared by mixing 0.5 g of Oil Red O in 100 mL of isopropanol and then vortexing it until homogenous. This was then mixed with ddH_2_O at a 3:2 ratio and filtered through a 0.22 μm syringe filter. After incubation, the dehydrated worms were centrifuged at 1500× *g* for 1 min and the supernatant discarded. Next, 200 μL of the Oil Red O working solution was added to each nematode tube, and each sample was incubated in the dark at 37 °C overnight. This was followed by centrifugation at 1500× *g* for 1 min, and the supernatant was discarded. From there, 1 mL of M9 + 0.1% Triton X-100 was added, followed by a 1 min 1500× *g* centrifugation, and the supernatant was again removed; this washing step was repeated twice. After the last wash, the worms were left in 100 μL of the M9 + 0.1% Triton X-100 solution.

The worms were then mounted on a glass slide upon a 2% agarose pad and analyzed under a ZEISS Axio Imager 2 using the Zeiss Axiocam 506 monochrome camera (ZEISS, Jena, Germany). The Zeiss LSM 710 Confocal Microscope, capable of fluorescence excitation using the Zeiss LSM non-descanned detector and creating an image with a digital Axiocam camera, was unable to be used in this assay due to the autofluorescence of the chlorophyll in the cyanobacteria diet interacting with the Oil Red O fluorescence at 488 nm fluorescence excitation, resulting in fluorescence quenching. As a result, in lieu of assessing fluorescence, a monochrome camera was used to assess Oil Red O dye uptake in the visible light spectrum. Intensity of the fat accumulation in each image was then measured at 100× magnification using Zeiss Zen Pro 2.3 image processing software.

### 5.5. Longevity Assay

Synchronized L1 worms were plated as described above, and a longevity assay was performed following a protocol modified from Park and colleagues [53] as follows. After 5 days of culturing at 20 °C, 10 worms per plate were transferred to a new plate with the same food source as they had previously. The same 10 worms per plate were then transferred every two days, and their survival was monitored, recorded, and analyzed. Worms were transferred to track their specific survival and to prevent confusion between them and their aging larval worm progeny.

### 5.6. Dauer Heat Stress Response Assay

Synchronized L1 worms were plated as described above, and a dauer assay was performed following a protocol modified from Zheng and colleagues [54]. Plates were cultured at 27 °C for 2 days, after which worms were inspected for dauer stress response, and the percent of worms in dauer per plate for each treatment was recorded.

### 5.7. Thrashing Assay

Synchronized L1 worms were plated as described above, and a thrashing assay was performed following a protocol modified from Nawa and Matsuoka [55]. Plates were cultured for 2 days at 20 °C, after which worms were picked and placed into 10 µL of M9. The worms’ movements were then filmed, and the amount of body thrashes in a 10 s period for each treatment was recorded. The rate of thrashes per minute was then calculated for each worm.

### 5.8. Pharyngeal Pumping Assay

Synchronized L1 worms were plated as described above and cultured at 20 °C for 4 days, and a pharyngeal pumping assay was performed following a protocol modified from Raizen and colleagues [56]. The worms in each treatment were then videoed for approximately 30 s at 400× magnification to record their pharyngeal pumping. The footage was then analyzed in 10 s increments where the pharynx was visible during feeding. The rate of pharyngeal pumps per min was then calculated for each worm per treatment.

### 5.9. SKN-1::GFP Expression

*C. elegans* LD1 obtained from Caenorhabditis Genetics Center was maintained on Nematode Growth Medium (NGM) agar plates [24] seeded with *E. coli* OP50 [48]. The Bristol wild-type strain N2 and the strain LD1 used in this study were cultured as described above for the N2 strain with toxic *M. aeruginosa* CPCC300, *E. coli* OP50, or *E. coli* OP50 with 30 mM sodium azide. The latter treatment is known to induce a stress response in the nematodes and acted as a positive control. The plates were cultured for 4 days at 20 °C and then imaged.

The worms were selected from their plates and immobilized using 0.10 µm Polybead Microspheres (Polysciences Inc., Warrington, PA, USA) in a 2.5% solid *w*/*v* aqueous suspension. The worms were then analyzed with a Zeiss LSM 710 Confocal Microscope (Oberkochen, Germany) at 100× magnification, and fluorescence was recorded under excitation at 488 nm wavelength [57].

### 5.10. Statistical Analysis

The results of the MC-LR assays were analyzed using the Tukey’s range test to determine the honest significant difference between treatments that had a significant *p*-value from their ANOVA with an α of 0.05 and a Q value from real-statistics.com [58]. The Q value was derived from the number of treatments (k) and the degrees of freedom (df) for each ANOVA; when the df value was not in the chart or between two values in the chart, the Q value with the higher df was chosen.

Two-tailed *t*-tests assuming homoscedastic variance were performed to analyze the results of fat accumulation, longevity, thrashing activity, and pharyngeal pumping.

Analysis of SKN-1::GFP expression was performed using single-factor ANOVAs to determine the statistical significance of the relative fluorescence intensity across each day, where the response variable is green fluorescence intensity, and the predictor variable is the treatment.

## Figures and Tables

**Table 1:** Physiological parameters assessed under different nutrient treatments: *M. aeruginosa* or *E. coli.* No significant differences between the diets were observed.

Physiological Parameter	*M. aeruginosa* X̄ ± SE	*E. coli* X̄ ± SE	n	*p*-Value
Dauer under Chronic Heat Stress (% in dauer)	86.27 ± 1.0	83.65 ± 1.0	8	0.10
Thrashing (thrashes/min)	209.25 ± 7.0	210.15 ± 4.4	40	0.91
Pharyngeal Pumping (pumps/min)	304.2 ± 9.3	330.0 ± 10.4	10	0.07

## Data Availability

The original contributions presented in this study are included in the article. Further inquiries can be directed to the corresponding author.
